# Supplementary material for: Causal Relationship between Aging and Anorexia Nervosa: A White-Matter-Microstructure-Mediated Mendelian Randomization Analysis
Source: Biomedicines. 2024 Aug 16;12(8):1874. doi: 10.3390/biomedicines12081874 (PMC11351342; doi:10.3390/biomedicines12081874)
Supplement: Supplementary file 1 [file biomedicines-12-01874-s001.zip › Supplementary Figure/Figure S4/Singe SNP plot of MR analysis for TL to ubm-b-1972.pdf]

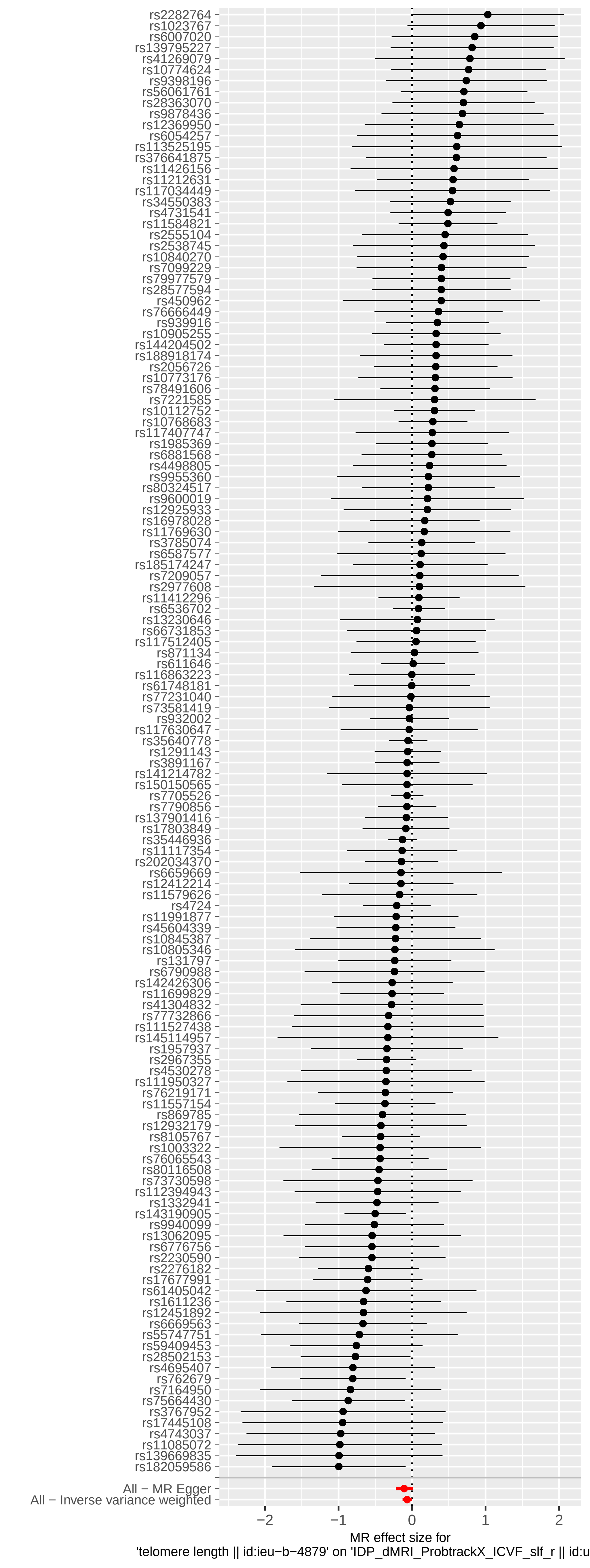
rs2282764
rs1023767
rs6007020
rs139795227
rs41269079
rs10774624
rs9398196
rs56061761
rs28363070
rs9878436
rs12369950
rs6054257
rs113525195
rs376641875
rs11426156
rs11212631
rs117034449
rs34550383
rs4731541
rs11584821
rs2555104
rs2538745
rs10840270
rs7099229
rs79977579
rs28577594
rs450962
rs76666449
rs939916
rs10905255
rs144204502
rs188918174
rs2056726
rs10773176
rs78491606
rs7221585
rs10112752
rs10768683
rs117407747
rs1985369
rs6881568
rs4498805
rs9955360
rs80324517
rs9600019
rs12925933
rs16978028
rs11769630
rs3785074
rs6587577
rs185174247
rs7209057
rs2977608
rs11412296
rs6536702
rs13230646
rs66731853
rs117512405
rs871134
rs611646
rs116863223
rs61748181
rs77231040
rs73581419
rs932002
rs117630647
rs35640778
rs1291143
rs3891167
rs141214782
rs150150565
rs7705526
rs7790856
rs137901416
rs17803849
rs35446936
rs11117354
rs202034370
rs6659669
rs12412214
rs11579626
rs4724
rs11991877
rs45604339
rs10845387
rs10805346
rs131797
rs6790988
rs142426306
rs11699829
rs41304832
rs77732866
rs111527438
rs145114957
rs1957937
rs2967355
rs4530278
rs111950327
rs76219171
rs11557154
rs869785
rs12932179
rs8105767
rs1003322
rs76065543
rs80116508
rs73730598
rs112394943
rs1332941
rs143190905
rs9940099
rs13062095
rs6776756
rs2230590
rs2276182
rs17677991
rs61405042
rs1611236
rs12451892
rs6669563
rs55747751
rs59409453
rs28502153
rs4695407
rs762679
rs7164950
rs75664430
rs3767952
rs17445108
rs4743037
rs11085072
rs139669835
rs182059586
All − MR Egger
All − Inverse variance weighted
−2
−1
0
1
2
MR effect size for
'telomere length || id:ieu−b−4879' on 'IDP_dMRI_ProbtrackX_ICVF_slf_r || id:u
